# The Proteome of the Dentate Terminal Zone of the Perforant Path Indicates Presynaptic Impairment in Alzheimer Disease*[Fn FN2]

**DOI:** 10.1074/mcp.RA119.001737

**Published:** 2019-11-07

**Authors:** Hazal Haytural, Georgios Mermelekas, Ceren Emre, Saket Milind Nigam, Steven L. Carroll, Bengt Winblad, Nenad Bogdanovic, Gaël Barthet, Ann-Charlotte Granholm, Lukas M. Orre, Lars O. Tjernberg, Susanne Frykman

**Affiliations:** ‡ Division of Neurogeriatrics, Center for Alzheimer Research, Department of Neurobiology, Care Sciences and Society, Karolinska Institutet, Solna, Sweden; § Department of Oncology-Pathology, Science for Life Laboratory, Karolinska Institutet, Stockholm, Sweden; ¶ Department of Neuroscience, Karolinska Institutet, Stockholm, Sweden; ‖ Department of Pathology and Laboratory Medicine, Medical University of South Carolina, Charleston, South Carolina; ** Karolinska University Hospital, Theme Aging, Stockholm, Sweden; ‡‡ Division of Clinical geriatrics, Center for Alzheimer Research, Department of Neurobiology, Care Sciences and Society, Karolinska Institutet, Huddinge, Sweden; §§ Interdisciplinary Institute for Neuroscience, CNRS UMR, Bordeaux, France; ¶¶ University of Bordeaux, Bordeaux, France; ‖‖ Knoebel Institute for Healthy Aging, University of Denver, Denver, Colorado

**Keywords:** Alzheimer's disease, mass spectrometry, clinical proteomics, immunohistochemistry, pathway analysis, neurodegenerative diseases, molecular layer of the dentate gyrus, perforant path, postmortem human brain, presynaptic impairment

## Abstract

Synaptic dysfunction is an early pathogenic event in Alzheimer disease (AD) that contributes to network disturbances and cognitive decline. Some synapses are more vulnerable than others, including the synapses of the perforant path, which provides the main excitatory input to the hippocampus. To elucidate the molecular mechanisms underlying the dysfunction of these synapses, we performed an explorative proteomic study of the dentate terminal zone of the perforant path. The outer two-thirds of the molecular layer of the dentate gyrus, where the perforant path synapses are located, was microdissected from five subjects with AD and five controls. The microdissected tissues were dissolved and digested by trypsin. Peptides from each sample were labeled with different isobaric tags, pooled together and pre-fractionated into 72 fractions by high-resolution isoelectric focusing. Each fraction was then analyzed by liquid chromatography-mass spectrometry. We quantified the relative expression levels of 7322 proteins, whereof 724 showed significantly altered levels in AD. Our comprehensive data analysis using enrichment and pathway analyses strongly indicated that presynaptic signaling, such as exocytosis and synaptic vesicle cycle processes, is severely disturbed in this area in AD, whereas postsynaptic proteins remained unchanged. Among the significantly altered proteins, we selected three of the most downregulated synaptic proteins; complexin-1, complexin-2 and synaptogyrin-1, for further validation, using a new cohort consisting of six AD and eight control cases. Semi-quantitative analysis of immunohistochemical staining confirmed decreased levels of complexin-1, complexin-2 and synaptogyrin-1 in the outer two-thirds of the molecular layer of the dentate gyrus in AD. Our in-depth proteomic analysis provides extensive knowledge on the potential molecular mechanism underlying synaptic dysfunction related to AD and supports that presynaptic alterations are more important than postsynaptic changes in early stages of the disease. The specific synaptic proteins identified could potentially be targeted to halt synaptic dysfunction in AD.

## Abstract (precis)

Synaptic dysfunction is an early pathogenic event in Alzheimer disease (AD). Hence the maintenance of healthy neurotransmission becomes crucial to slow or halt cognitive decline. In search of identifying proteins that could play a role in synaptic dysfunction, we studied the proteome of a highly vulnerable hippocampal region that is enriched in excitatory synapses. Our in-depth proteomic analysis suggests an impaired presynaptic signaling in AD. Using immunohistochemistry, we verified significantly reduced levels of complexin-1, complexin-2, and synaptogyrin-1 in AD.

## Graphical Abstract

**Figure:**
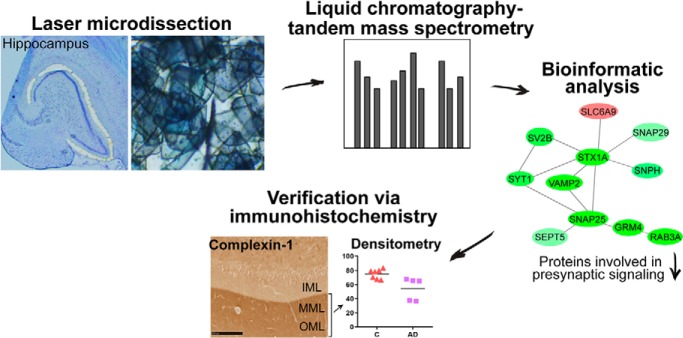

**Highlights**

- Laser microdissection of highly vulnerable hippocampal region.
- Proteomic analysis of postmortem human brain tissue of AD and control cases.
- Decreased levels of presynaptic proteins, but not postsynaptic proteins, in AD.
- Immunohistochemistry verifies decreased levels of selected presynaptic proteins.

Alzheimer disease (AD)^1^ is a progressive neurodegenerative disorder and the most common cause of dementia that affects 50–60% of all dementia cases. AD is characterized by its underlying neuropathological process including the accumulation of amyloid plaques that are composed of amyloid β-peptide (Aβ), and neurofibrillary tangles consisting of hyperphosphorylated tau protein, and neurodegeneration (1). Over the past decades, substantial progress has been made in elucidating potential mechanisms underlying AD pathogenesis including mitochondrial dysfunction (2), autophagy (3), excitotoxicity (4), and inflammation (5). However, there is still an urgency to decipher early pathogenic processes in order to develop disease-modifying therapeutics. Recently, more attention has been devoted to synaptic dysfunction, because synapse loss and decreased levels of synaptic proteins in AD brains strongly correlate with the degree of cognitive decline (6–8). However, the molecular mechanisms underlying the synaptic dysfunction are largely unknown.

The hippocampal formation, consisting of the cornu ammonis (CA) regions of the hippocampus, the dentate gyrus (DG) and the subiculum, plays a crucial role in episodic memory formation and spatial learning (9). The highly laminated entorhinal cortex provides the major excitatory inputs to the hippocampus through the perforant path (Fig. 1*A*) (10). The axons of the perforant path mainly arise from the layer II and III entorhinal neurons. Although axons from layer II terminate primarily on the dendritic spines of granule cells located strictly in the outer two-thirds of the molecular layer (ML) of the DG, those arising from layer III terminate on the dendrites of CA1 pyramidal neurons. Extensive research has shown i) the presence of amyloid plaques and neurofibrillary tangles both in the entorhinal cortex and in the ML of the DG (11, 12), ii) dramatic loss of entorhinal neurons, especially in the layer II (13, 14), and iii) decreased number of synapses in the outer ML of the DG in AD brains (15, 16). These findings suggest that AD-related pathologic alterations could damage the connectivity between the hippocampal formation and entorhinal cortex, and therefore, could contribute to cognitive impairment. Elucidating the mechanisms underlying this disconnectivity is therefore of utmost importance.

**Fig. 1.**
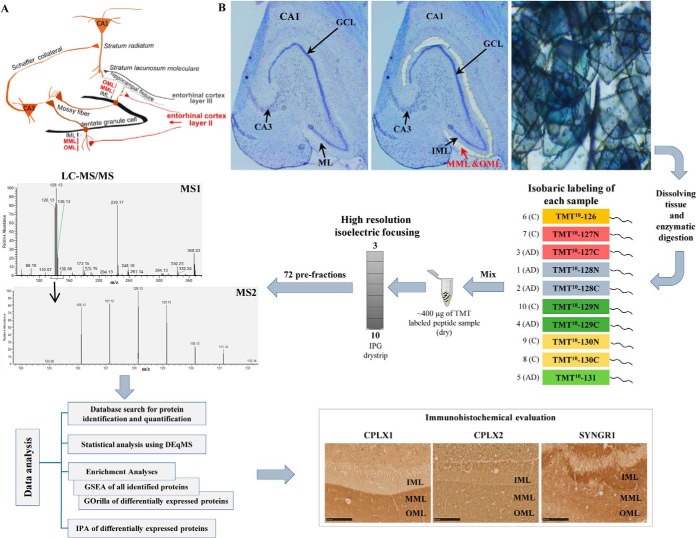
**Overview of experimental workflow of the study.** *A*, A schematic illustration of the major hippocampal circuits. The molecular layer (ML) of the dentate gyrus (DG) consists of inner (IML), middle (MML) and outer molecular layer (OML). The perforant path provides the main excitatory input of the hippocampus. The fibers of the layer II perforant path terminate in the outer two-thirds of the ML of the DG (highlighted in red). *B*, The MML and OML of the DG was isolated from five sporadic AD and five control cases using laser microdissection. The microdissected tissue was then dissolved, digested, and the resulting peptides from the ten samples were labeled with ten different isobaric tags (TMT10plex 126–131Da), and pre-fractionated into 72 fractions using HiRIEF with the broad range pH 3–10 IPG drystrip. Each fraction was analyzed by LC-MS/MS. DEqMS was used for statistical analysis and proteins were further subjected to different enrichment and pathway analysis tools, using GSEA, GOrilla and IPA. Lastly, the expression of three proteomic hits (CPLX1, CPLX2, and SYNGR1) was confirmed in a new cohort consisting of five AD and seven control cases using immunohistochemistry.

Functional synapses are crucial for formation of memory and learning. Synaptic transmission requires a series of interactions between synaptic vesicle (SV) proteins, presynaptic and postsynaptic membrane proteins (17, 18). To date, a number of synaptic proteins involved in presynaptic (RAB3A, SNAP25, septin-5, SV2A, synapsin I, synaptophysin, synaptotagmin, syntaxin-1a) and postsynaptic mechanisms (drebrin, neurogranin, NMDA and AMPA receptors, PSD95, synaptopodin) has been found to be altered in AD brains (19, 20) - emphasizing that synaptic integrity is diminished in AD.

Mass spectrometry (MS)-based proteomics has a great potential to investigate changes in protein expression in an unbiased manner, and therefore, could lead to identification of proteins underlying disease mechanisms. Several research groups have applied mass spectrometry to elucidate changes in the proteome of different brain regions including the hippocampus (21–25). Additionally, a few studies have investigated the proteome of the hippocampal subfields that were microdissected from AD brains, such as CA1 pyramidal neurons (26), CA4 (27), CA1 and subiculum (28). However, to our knowledge, no proteomic study has been performed aiming to gain a better understanding of the synaptic dysfunction of the vulnerable perforant path synapses. In order to reach this goal, we microdissected the outer two-thirds of the ML of the DG, where these synapses are located, and performed state-of-the-art mass spectrometry. Our in-depth proteome analysis resulted in an identification of many synaptic proteins that shows altered expression levels in AD brains. Interestingly, there was an over-representation of presynaptic proteins among the decreased proteins. For three of these proteins, complexin-1 (CPLX1), complexin-2 (CPLX2) and synaptogyrin-1 (SYNGR1), the decreased expression in the outer two-thirds of the ML of the DG in AD was confirmed in another cohort of AD and control cases, using immunohistochemical staining.

## EXPERIMENTAL PROCEDURES

### 

#### 

##### Experimental Design and Statistical Rationale

**Fig. 2.**
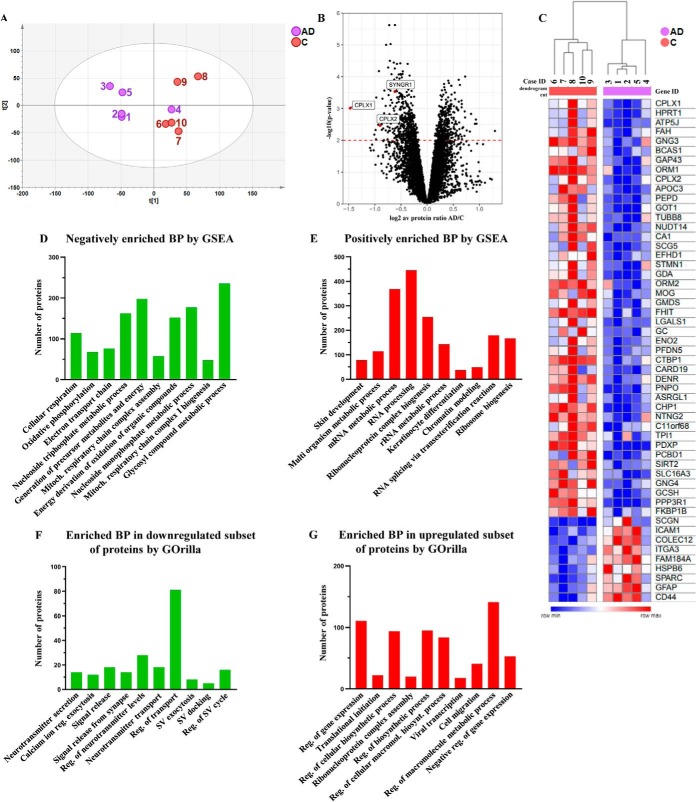
**The proteomic profile of the terminal zone of the perforant path in AD.** *A*, Principal component analysis identified two main clusters, which can be defined by clinical diagnosis, except AD case 4. *B*, A volcano plot showing the changes in the protein expression levels between AD and control groups. Proteins with increased levels in AD are shown on the positive side of the *x* axis, whereas decreased proteins are shown on the negative side of the *x* axis. Y-axis shows the statistical significance. Hits above the red dashed line corresponds to differentially expressed proteins with *p* < 0.01 and FDR <10%. *C*, The most altered proteins (log_2_ fold change −/+ 0.7; corresponding to more than 38% decrease or 64% increase, and FDR <10%) are shown in the heat map. *D–E*, All of the identified proteins were sorted by their fold change and analyzed using GSEA regardless of their p- and FDR-values. The top 10 negatively (*D*) and positively (*E*) enriched biological processes (BPs) in AD were plotted. GO terms with *p* < 0.01 and FDR <5% were considered statistically significant. *F–G*, The significantly decreased or increased proteins (FDR <10%, target lists) were compared with the entire dataset (background list) by GOrilla. The top 10 enriched BPs in the downregulated (*F*) and up-regulated (*G*) subset of proteins are shown. GO terms with FDR <5% were considered statistically significant.

This study consists of two stages in which we first studied the proteome of the outer two-thirds of the ML of the DG in the exploratory cohort of five AD and five neurologically healthy control cases using an unbiased MS approach, and then performed an immunohistochemical analysis of three identified proteins in the verification cohort of an additional five AD and seven neurologically healthy control cases. The relatively low number of biological replicates used in the proteomics study were chosen because of the extensive work-load required to perform laser microdissection (LMD), and the aim to reach as deep as possible into the proteome through extensive fractionation performed with the high resolution isoelectric focusing (HiRIEF) protocol (72 fractions) before liquid chromatography-mass spectrometry (LC-MS). No technical replicates were performed, primarily because of very limited amount of clinical material, but also as our previous experience show very low technical variability between MS runs using HiRIEF LC-MS. The validity of the cohort was, however, confirmed by the low variability between samples and the fact that AD and control samples separated well in a principal component analysis (PCA) (Fig. 2*A*).

The hypothesis tested for each protein was that they were differentially expressed in AD samples compared with control samples. As we cannot assume normal distribution in the data using Shapiro-Wilk test, a non-parametric statistical method, DEqMS (Differential Expression analysis of quantitative Mass Spectrometry data, Zhou *et al.*, manuscript under consideration for publication elsewhere, R package in Bioconductor) was used. DEqMS works on top of limma package and considers the detected number of peptide spectra matches (PSMs) per peptide while calculating t-statistics. Benjamini-Hochberg method was used for correction for multiple hypothesis testing. An overview of the experimental workflow is shown in Fig. 1. The details of the experimental design and statistical rationale are explained in each subsection.

##### Case Selection

Frozen hippocampal tissues were obtained from five sporadic AD and five control cases from the Netherlands Brain Bank (Exploratory cohort). AD cases were clinically diagnosed according to previously published research criteria (29), and definitive diagnosis was confirmed pathologically by using immunohistochemical staining against amyloid plaques and neurofibrillary tangles. We selected AD cases with relatively early stages of AD pathology (AD Braak stage 4 and amyloid deposits stage C). Control cases showed no sign of neurological or psychiatric disorders and presented little or no pathological alterations beyond normal age-appropriate changes including a few plaques and tangles (AD Braak stage 0–2 and amyloid deposits stage 0-B). For immunohistochemical analysis, we obtained a different set of paraffin-embedded hippocampal sections from five AD and seven control cases from the Carroll A. Campbell Jr. Neuropathology Laboratory (CCNL) Brain Bank at the Medical University of South Carolina (Verification cohort). Each of these cases in the verification cohort had undergone a full neuropathological diagnosis including Braak and CERAD assessments (30). All cases in the respective cohorts were used throughout the studies. Demographic characteristics of all cases used in this study are shown in supplemental Table S1. All donors or their next-of-kin gave informed consent. This study was approved by the Regional Ethical Review Board in Stockholm (2015/18/03–31/02) and had also obtained Institutional Review Board approvals by the VU Medical Center and the Medical University of South Carolina.

##### Preparation of Tissue Samples for LMD

Consecutive sections (20 μm) were cut from the frozen hippocampal tissues using a cryostat (Leica Microsystems, Wetzlar, Germany) and mounted on polyethylene naphthalate membrane coated slides (1.0 PEN(D), Zeiss, Oberkochen, Germany). Slides were air-dried for 5 min, fixed in 75% ethanol for 1 min and stained with toluidine blue (Sigma-Aldrich, St. Louis, Missouri) for 1 min. Slides were rinsed with distilled water, air-dried and finally stored at −20 °C overnight. Toluidine blue stains the nucleus of all types of cells and allowed us to identify the ML of the DG which is located between the dentate granule cell layer and the hippocampal fissure. The outer two-thirds of the ML, covering the middle and the outer ML, was microdissected using LMD 6000/7000 (Leica Microsystem). To obtain enough amount of tissue for downstream proteomics approach, ∼0.6 mm^3^ of microdissected tissues were collected from 20–30 sections per case. No obvious anatomical differences were observed between the first and the last section during LMD. Finally, the microdissected tissues were stored at −20 °C until further use.

##### Sample Preparation for Mass Spectrometry

The microdissected tissue was dissolved in SDS-lysis buffer (4% SDS, 25 mm Hepes pH 7.6, 1 mm DTT). Lysates were heated at 95 °C for 5 min and then sonicated for 1 min to shear DNA. Samples were centrifuged at 13,000 × *g* to remove cell debris. The supernatant was collected and alkylated with 4 mm chloroacetamide. A modified Sera-Mag SP3 protocol was performed for sample clean-up (31). The SP3 beads-protein mixture was digested first by Lys-C (Pierce, Thermo Scientific, Waltham, Massachusetts) for 16 h prior to trypsin digestion (16 h). Finally, the peptides were eluted from the beads-protein mixture. Peptide concentration was measured by DC-protein assay (Bio-Rad, Hercules, California), and 45 μg of peptides from each sample were labeled with ten different amine-reactive isobaric tandem mass tags (TMTs) (TMT10plex 126–131Da, Thermo Scientific). An aliquot of ∼2 μg was suspended in LC mobile phase A and 1 μg was injected on the LC-MS to determine the labeling efficiency for each TMT-tag. Finally, the sample were pooled, and sample clean-up was applied by solid phase extraction (SPE strata-X-C, Phenomenex, Torrance, California), and purified samples were dried in a SpeedVac.

##### HiRIEF Separation

The pre-fractionation was done using HiRIEF (32). The pooled sample (450 μg) was dissolved in 250 μl of 8 m urea and 1% IPG pharmalyte (broad range pH 3–10, GE Healthcare, Chicago, Illinois), and the IPG drystrip was rehydrated overnight. The peptides were focused on the gel strip based on their isoelectric point and eluted into 72 contiguous fractions, as described previously (24).

##### Mass Spectrometry

For each LC-MS run of a HiRIEF fraction, the auto-sampler (UltiMate™ 3000 RSLCnano System, Thermo Scientific Dionex) dispensed 20 μl of mobile phase A (95% water, 5% DMSO, 0.1% formic acid) into the corresponding well of the microtitre plate, and 10 μl were injected to the LC-MS. Samples were trapped on a C18 guard-desalting column (Acclaim PepMap 100, 75 μm × 2 cm, nanoViper, C18, 5 μm, 100Å), and separated on a 50 cm long C18 column (Easy spray PepMap RSLC, C18, 2 μm, 100Å, 75 μm × 50 cm). At a constant flow of 250 nl/min, the curved gradient went from 2% mobile phase B (5% water, 5% DMSO, 95% acetonitrile, 0.1% formic acid) up to 40% solvent B in each fraction as shown in the supplemental Table S2, followed by a steep increase to 100% solvent B in 5 min. Online liquid chromatography-tandem mass spectrometry (LC-MS/MS) was performed using a hybrid Q-Exactive-HF mass spectrometer (Thermo Scientific). FTMS master scans with 70,000 resolution (and mass range 300–1700 mass to charge ratio (m/z)) were followed by data-dependent MS/MS (35 000 resolution) on the top five ions using higher energy collision dissociation at 30–40% normalized collision energy. Precursors were isolated with a 2 *m*/*z* window. Automatic gain control targets were 1 × 10^6^ for MS1 and 1 × 10^5^ for MS2. Maximum injection times were 100 ms for MS1 and 150–200 ms for MS2. The entire duty cycle lasted ∼2.5 s. Dynamic exclusion was used with 60 s duration. Precursors with unassigned charge state or charge state 1 were excluded. An underfill ratio of 1% was used.

##### Peptide and Protein Identification

Orbitrap raw MS/MS files were converted to mzML format using msConvert from the ProteoWizard tool suite (33). Spectra were then searched using MSGF+ (v10072) (34) and Percolator (v2.08) (35), where search results from eight subsequent fractions were grouped for Percolator target/decoy analysis. All searches were done against the human protein subset of Ensembl 75 in the Galaxy platform, including 51,153 entries (36). MSGF+ settings included precursor mass tolerance of 10 ppm, fragment ion mass tolerance of 0.02 Da, fully-tryptic peptides, maximum peptide length of 50 amino acids, a maximum charge of 6 and maximum 2 missed cleavages. Fixed modifications were TMT10plex on lysine residues and peptide N termini, and carbamidomethylation on cysteine residues, a variable modification was used for oxidation on methionine residues. Quantification of TMT10plex reporter ions was done using OpenMS project's IsobaricAnalyzer (v2.0) (37). PSMs found at 1% false discovery rate (FDR) were used to infer gene identities. Protein quantification was based on TMT10plex reporter ions and calculated using TMT PSM ratios to the entire sample set (all 10 TMT-channels) and normalized to the sample median. The median PSM TMT reporter ratio from peptides that are unique to a gene symbol was then used for quantification. Only one unique peptide was required to identify a protein, and a protein FDR cut-off of 1% was applied to the list of gene-centric proteins using the picked-FDR method (38). Peptide sequences, *m*/*z*, modifications, peptide identification scores, accession numbers, number of peptides, percentage coverage and quantification measurements for each peptide and protein has been deposited to the ProteomeXchange Consortium using the PRIDE repository (dataset identifier: PXD014557). Proteins that were not identified in all 10 cases were excluded in the analysis.

##### Statistical Analysis

Alterations in average protein expression between AD and control cases were calculated using DEqMS package in R (Zhou *et al.*, manuscript under consideration for publication elsewhere), because the data did not show normal distribution using Shapiro-Wilk test. DEqMS works on top of limma package and considers the detected number of PSMs per peptide while calculating t-statistics. DEqMS is available at Bioconductor (39), and the package is also deposited at GitHub (https://github.com/yafeng/DEqMS). Benjamini-Hochberg method was used for multiple hypothesis testing and a cut-off level of 10% FDR was applied.

Multivariate data analyses were performed to find the biggest variation in our dataset using an unsupervised PCA in SIMCA v15. A heat map of differentially expressed proteins (log_2_ fold change −/+ 0.7 and FDR <10%) was generated using Morpheus (Broad Institute) and hierarchical clustering with complete linkage based on Pearson distance metrics was applied to visualize the expression profile between AD and control cases. To compare demographic characteristics (including age, gender and postmortem interval (PMI), pH and ApoE) between AD and control groups, we did univariate analyses using Mann Whitney test in GraphPad PRISM 7.0 (San Diego, CA). Statistical analysis of semi-quantitative densitometry assessment of immunohistochemical staining was carried out using Mann Whitney test in GraphPad PRISM 7.0 (San Diego, CA).

##### Bioinformatics Analysis

##### Enrichment Analyses

To have an overview of which biological processes might be affected in the outer two-thirds of the ML of the DG in AD, we used gene set enrichment analysis (GSEA, Broad Institute) (40). This type of analysis is based on the molecular signature database (v6.1) that contains gene sets derived from gene ontology (GO) annotations (41). We ran a running-sum statistic method, in which all of the identified genes were ranked from the most increased to the most decreased expression regardless of their p- and FDR-values (GO database from 16–02-2019). Ontologies with *p* < 0.01 and FDR <5% were considered significantly enriched which is considerably more stringent than the GSEA guideline (40). Additionally, we performed a second type of enrichment analysis using the GOrilla tool (GO database from 27–04-2019) (42). Two unranked lists of proteins, a target list consisting of differentially expressed proteins (FDR <10%) and a background list, which is all the identified proteins, were uploaded. We performed this analysis for decreased and increased proteins separately. Ontologies with FDR <5% were considered statistically significant. Both in GSEA and GOrilla tools, Benjamini-Hochberg method was used to calculate FDR-values.

##### Pathway Analyses

Ingenuity pathway analysis (IPA, Qiagen, Hilden, Germany) was used for systemic analysis of our dataset in order to interpret our findings in a biological context. AD/C protein ratio (log_2_) of all quantified proteins, p- and FDR-values were uploaded into the IPA, and core analysis was performed. We allowed both direct and indirect relationships and a search on published data carried out in mouse, rat and human species, and applied 10% FDR cut-off to avoid having relations that could occur just by chance. Differentially expressed proteins were then analyzed in three categories: biological functions, enriched molecular networks, and upstream regulators. For each biological function and upstream regulator, IPA calculates a *p* value using right-tailed Fisher's exact test and *p* < 0.01 is considered statistically significant. Moreover, IPA makes a prediction about the activation state by calculating a z-score, which are considered significant if z-score >2 or z-score <−2. Lastly, IPA can create molecular networks based on the identified proteins, and for each network, IPA assigns a score that is calculated from hypergeometric distribution and right-tailed Fisher's Exact test.

##### Immunohistochemistry

Paraffin-embedded hippocampal sections (5 μm) were rehydrated and heat-induced epitope retrieval was applied using citrate buffer (pH 6) or Tris-EDTA buffer (pH 9) at 110 °C for 30 min. EnVision Dual Link System (Dako, Agilent, Santa Clara, California) containing peroxidase, HRP-labeled polymer, DAB and DAB substrate buffer, was used to visualize DAB staining. After blocking endogenous peroxidase activity for 5 min, sections were blocked with goat serum (Dako, Agilent) for 30 min at room temperature, and then stained with the following primary antibodies overnight at 4 °C: complexin-1 (CPLX1, Protein Tech, Rosemont, Illinois, 1:200 dilution), complexin-2 (CPLX2, Protein Tech, 1:200 dilution), and synaptogyrin-1 (SYNGR1, Sigma-Aldrich, 1:200 dilution). Antibody dilutions were prepared in antibody diluent (Dako, Agilent). Sections were then incubated with HRP-labeled polymer for 1 h at room temperature, followed by incubating with DAB. Finally, sections were dehydrated and coverslipped using VectaMount Permanent Mounting Medium (Vector Laboratories, Burlingame, California). Sections from AD and control groups were included in the same staining experiment and using the same antibody solutions to avoid inter-staining variability. Sections were scanned using Nikon Eclipse E800 microscope and Nikon DS-Ri2 camera (10× objective, gain 1, exposure 2 ms) and NIS Elements software (Nikon Instrument, Inc., Japan), and images were analyzed on 8-bit scale using ImageJ Fiji (NIH). Mean pixel intensities of the area of interest, covering the middle and outer ML of the DG, were measured in a blinded fashion and the background, defined as the signal intensity of a region outside the tissue, was subtracted (43). Additionally, gross morphological observations of the staining intensity in the region of interest was checked by subjective assessment done by two independent researchers (C.E. and S.F.) in a blinded fashion. Visual scoring was performed based on a scale from 0 to 3 (0, absent; 1, mild; 2, moderate; 3, strong).

## RESULTS

### 

#### 

##### Demographic Characteristics

Demographic characteristics of all cases used in this study are shown in supplemental Table S1. We used one cohort from the Netherlands Brain Bank for the initial proteomic study (exploratory) and another cohort from the Carroll A. Campbell Jr. Neuropathology Laboratory (CCNL) Brain Bank for the immunohistochemistry study (verification). When using end-stage material, variability between the cases is inevitable, and therefore, it is crucial to have a well-characterized study cohort. We tried to control this variability as much as possible by considering age, gender, PMI, and AD-related pathology. Moreover, in order to minimize the effect of synapse loss, we selected AD cases with relatively early stages of AD (AD Braak stage 4 and amyloid deposits stage C) for the exploratory cohort. There were no statistically significant differences in age, gender or PMI between the groups in either of the cohorts. The pH of the brain tissue showed no significant difference between the groups in the exploratory cohort. For the verification cohort, all of the AD cases but none of the control cases were ApoE4 carriers, in line with ApoE4 being a risk factor for AD.

##### Identification of Differentially Expressed Proteins

To identify proteins that may play a role in synaptic dysfunction, we studied the proteome of the outer two-thirds of the ML of the DG, because excitatory synapses of the perforant path-granule cells are highly enriched in this region. In addition, this region also contains, to a lesser extent, axons and dendrites derived from various cell types, glial cells and inhibitory MOPP (molecular layer perforant path-associated) cells. Considering the fact this region is highly vulnerable to AD-related pathologic changes, we used LMD to dissect it from five AD and five neurologically healthy control cases and performed quantitative MS. All cases were labeled with a different isobaric tag on a peptide level, allowing simultaneous detection of ten samples, and thereby, eliminating variation between runs. Prior to LC-MS/MS, labeled peptides were fractionated into 72 fractions based on their isoelectric focusing point (32). Our in-depth proteomic analysis led to an identification of 68,755 peptides mapping to 7322 proteins (gene-centric) using the MSGF+ Percolator software (1% FDR cut-off in peptide level). Protein false discovery rates were calculated using the picked-FDR method using gene symbols as protein groups and limited to 1% FDR (38). The complete list of proteins that were quantified in all cases can be found in supplemental Table S3. Quantitative information about both peptide and protein level has also been deposited to the ProteomeXchange Consortium using the PRIDE repository (dataset identifier: PXD014557). PCA shows a relatively good separation between AD and control cases, except for one of the AD cases, ID-4 (Fig. 2*A*). The clinical and neuropathological details of this AD patient do not suggest any prominent evidence that could explain this discrepancy. The distribution of fold change *versus p* values for all identified proteins is shown in a volcano plot displaying a considerable change in the protein levels between AD and control (Fig. 2*B*). After adjusting for multiple hypothesis comparison using Benjamini-Hochberg method, 724 proteins were found to be significantly altered (*p* < 0.01 and FDR <10%), consisting of 382 decreased and 342 increased proteins. The expression levels of the most altered proteins (FDR <10%) are shown in the heat map (Fig. 2*C*), and the top 20 proteins with the largest fold change are listed in Table I. We observed an over-representation of presynaptic proteins among the decreased proteins including complexins, RAB3A, metabotropic glutamate receptor 4 (GRM4) and SYNGR1. Interestingly, however, postsynaptic proteins such as AMPA and NMDA receptors, drebrin, PSD95, HOMER1 and synaptopodin were not altered in AD (supplemental Table S4). There was no difference in the levels of amyloid precursor protein (APP) and microtubule-associated protein tau (MAPT) between AD and control cases in our dataset. However, this is not surprising, because even if the region of interest contains amyloid plaques and neurofibrillary tangles, they were most probably not dissolved using the current methodology. In addition, the fact that the total amount of APP and tau were not altered does not rule out the possibility that the levels of Aβ or phosphorylated tau were altered.

**Table I:** The top 10 decreased and top 10 increased proteins showing the largest changes in AD

Gene name	Protein name	log_2_ of av. ratio (AD/C)	ratio (AD/C)	PSM count	*p* value	Adjusted *p* value (FDR)	Biological process
CPLX1	Complexin-1	−1.47	0.36	10	0.00098	0.06	Exocytosis, Neurotransmitter transport, Transport
HPRT1	Hypoxanthine-guanine phosphoribosyltransferase	−1.01	0.50	14	0.00663	0.08	Purine salvage pathway, Inosine monophosphate metabolic process
ATP5J	ATP synthase-coupling factor 6, mitochondrial	−0.99	0.50	8	0.00477	0.08	Hydrogen ion transport, Ion transport, Transport
FAH	Fumarylacetoacetase	−0.98	0.51	5	0.00932	0.09	Phenylalanine catabolism, Tyrosine catabolism
GNG3	Guanine nucleotide-binding protein G(I)/G(S)/G(O) subunit gamma-3	−0.97	0.51	6	0.00045	0.05	G protein-coupled receptor signalling pathway
BCAS1	Breast carcinoma-amplified sequence 1	−0.94	0.52	19	0.00324	0.07	Myelination
GAP43	Neuromodulin	−0.93	0.53	268	0.00181	0.06	Regulation of growth
ORM1	Alpha-1-acid glycoprotein 1	−0.92	0.53	7	0.00007	0.04	Inflammatory response
CPLX2	Complexin-2	−0.90	0.54	17	0.00343	0.07	Exocytosis, Neurotransmitter transport, Neurogenesis
APOC3	Apolipoprotein C-III	−0.89	0.54	1	0.00200	0.06	Lipoprotein metabolic process, Cholesterol homeostasis
HRC	Sarcoplasmic reticulum histidine-rich calcium-binding protein	1.30	2.46	2	0.00510	0.08	Cellular protein metabolic process, muscle contraction
JPH2	Junctophilin-2	0.94	1.92	1	0.00300	0.07	Calcium ion homeostasis
CD44	CD44 antigen	0.93	1.91	48	0.00222	0.06	Cell adhesion, Inflammatory response, Extracellular matrix organization
GFAP	Glial fibrillary acidic protein	0.92	1.89	2445	0.00304	0.07	Astrocyte development
SPARC	SPARC	0.84	1.79	6	0.00148	0.06	Extracellular response to growth factor stimulus, Extracellular matrix organization, Response to calcium ion, Regulation of synapse organization
HSPB6	Heat shock protein beta 6	0.82	1.76	5	0.00531	0.08	Chaperone-mediated protein folding
FAM184A	Protein FAM184A	0.76	1.69	28	0.00490	0.08	–
ITGA3	Integrin alpha 3	0.73	1.66	5	0.00010	0.04	Extracellular matrix organization, Cell-matrix adhesion, Dendritic spine maintenance, Memory
COLEC12	Collectin 12	0.73	1.66	4	0.00191	0.06	Regulation of immune response
ICAM1	Intercellular adhesion molecule 1	0.72	1.65	11	0.00123	0.06	Cell adhesion, Regulation of immune response

##### Enrichment Analyses Indicate Altered Mitochondrial and Synaptic Function

We used GSEA in order to get an overview of the biological processes that might be affected in the outer two-thirds of the ML of the DG by AD pathogenesis. This analysis considered the observed fold change of all identified proteins and resulted in 94 negatively and 118 positively enriched GO terms (*p* < 0.01 and FDR <5%, supplemental Table S5 and 6). The most negatively and positively enriched 10 biological processes ranked by *p* value are shown in Fig. 2*D* and 2*E*, respectively, and individual enrichment plots can be found in the supplemental Fig. S1 and S2. The negative enrichment of processes including cellular respiration, oxidative phosphorylation and electron transport chain clearly indicate altered energy metabolism in the region of interest in AD. In addition, calcium ion regulated exocytosis and SV localization were also found to be negatively enriched in AD (supplemental Table S5). On the other hand, among the top 10 positively enriched GO terms, RNA processing and ribonucleoprotein complex biogenesis were more prominent (Fig. 2*E*).

Additionally, using the GOrilla tool, we investigated the enrichment of biological processes when only the differentially expressed proteins (FDR <10%) (target list) were compared with all identified proteins in our dataset (background list). Enrichment analysis performed only in decreased proteins (*n* = 382, FDR <10%) resulted in 55 GO terms (FDR <5%, supplemental Table S7). The most enriched biological processes in this subset include neurotransmitter secretion, calcium ion regulated exocytosis, and signal release (Fig. 2*F*). On the other hand, enrichment analysis of increased proteins (*n* = 342, FDR <10%) identified 92 enriched GO terms (FDR <5%, supplemental Table S8). Like GSEA findings, biological processes related to gene expression seemed to be positively enriched in GOrilla (Fig. 2*G*).

##### Pathway Analyses of Differentially Expressed Proteins

**Fig. 3.**
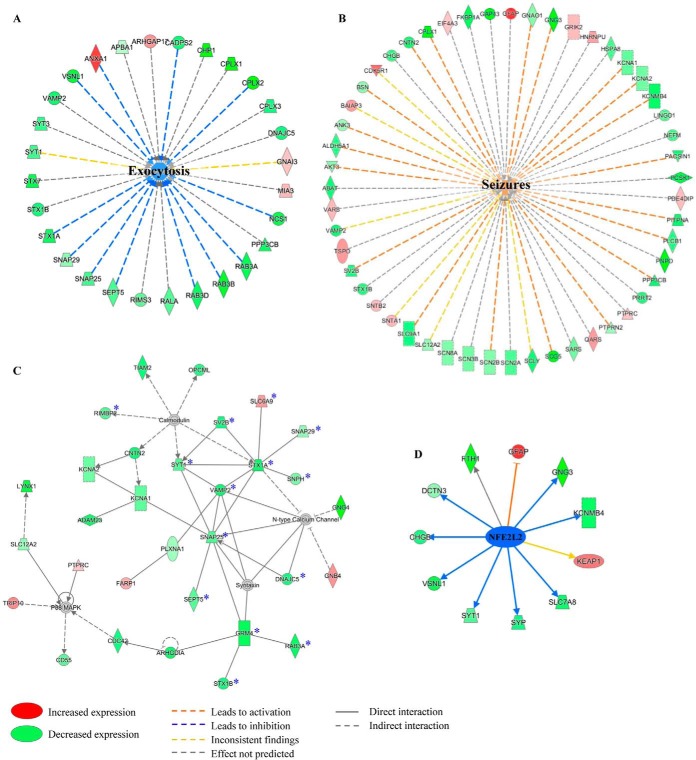
**Pathway analyses of significantly altered proteins by IPA.** Different IPA tools were used to get a better understanding of biological processes that are affected in AD. *A–B*, The diseases and functions tool was used to predict biological functions affected by the differentially expressed proteins. Exocytosis was predicted to be decreased (*A*), whereas seizures was predicted to be increased in AD (*B*). *C*, The molecular networks tool generated 25 different networks. The network “Cell-to-cell signaling and interaction/Nervous system development and function/Cellular assembly and organization”, shown in (*C*), was ranked with the highest score and consisted of pathways mediating neurotransmission. An asterisk indicates presynaptic proteins in this network. *D*, The upstream regulator tool predicted the transcription factor Nuclear factor erythroid 2-related factor 2 (NFE2L2) as an upstream regulator of its target proteins that were identified in our dataset.

To further illuminate the biological activities occurring in the outer two-thirds of the ML of the DG in AD, we performed a detailed pathway analysis on differentially expressed proteins (*n* = 724, FDR <10%) using IPA, which predicts affected biological processes, molecular networks and upstream regulators based on literature. First, the diseases and functions tool was used to identify key biological processes influenced by the observed expression profile in the region of interest (supplemental Table S9). For instance, IPA predicted that exocytosis was decreased in AD because of the expression profile of annexin-A1, calcium-dependent secretion activator 2 (CADPS2), CPLX2, neuronal calcium sensor-1 (NCS1), RAB3A, RAB3B, RAB3D, septin-5, SNAP25, SNAP29, syntaxin-1a, and visinin-like protein 1 (VSNL1) (Fig. 3*A*). Other predicted decreased processes include secretory pathway, neuron migration, migration of central nervous system cells and transport of metal ion. In addition, IPA predicted an increase of several pathways including seizures (Fig. 3*B*), cell spreading of tumor cell lines, ataxia, shape change of tumor cell lines, phosphorylation of proteins, tremor, and cell survival (supplemental Table S9). Secondly, IPA can create molecular networks that can provide more information about the relationship between the identified proteins. A total of 25 molecular networks were created and a network called “cell-to-cell signaling and interaction/nervous system development and function/cellular assembly and organization” was found to be affected the most in AD (Fig. 3*C*). Interestingly, this network consists of pathways mediating neurotransmission (*p* = 4.92E-13), synaptic transmission (*p* = 2.24E-11), transport of SVs (*p* = 3.74E-10), and release of neurotransmitter (*p* = 7.46E-09). Lastly, the upstream regulator tool was used to identify which upstream molecules can potentially explain the observed changes in protein expression in our dataset. MAP kinase-interacting serine/threonine-protein kinase 1 (MKNK1), transcription factor 7-like 2 (TCF7L2), hemoglobin subunit alpha (HBA1/HBA2), and nuclear factor erythroid 2-related factor 2 (NFE2L2) were identified as upstream regulators and all predicted to be inhibited with significant z-scores (supplemental Table S10). Notably, Nrf2 (NFE2L2) is a key regulator of the antioxidant metabolism and has been extensively studied in the field of neurodegenerative disorders (Fig. 3*D*). Interestingly, APP and MAPT were also identified as upstream regulators but activation state could not be predicted. Intriguingly, IPA did not identify any significant activated upstream regulators.

##### Immunohistochemical Analysis

**Fig. 4.**
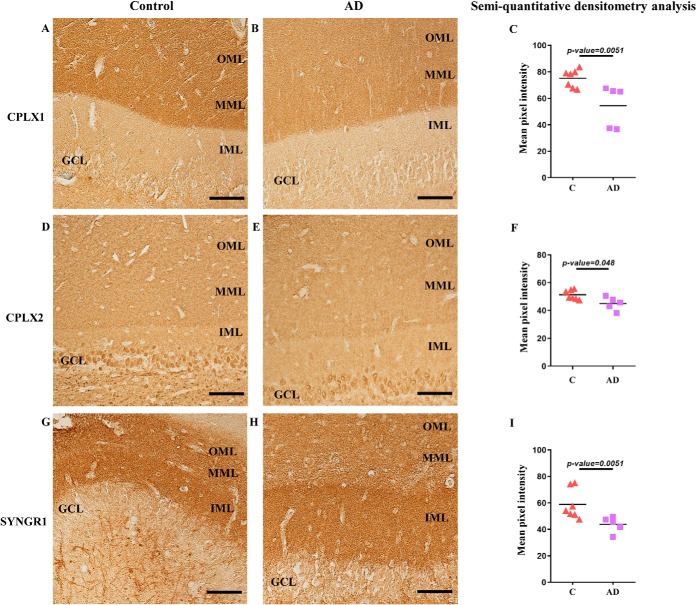
**Immunohistochemical analysis of selected proteomic hits.** Immunohistochemistry was used to validate the alterations for CPLX1 (*A–C*), CPLX2 (*D–F*), and SYNGR1 (*G–I*) in seven control and five AD cases. Representative figures of one control case (*A*, *D*, and *G*) and one AD case (*B*, *E*, and *H*) are shown. Densitometry measurements were performed using Image J and mean pixel intensities are shown. Semi-quantitative densitometry analysis showed significantly decreased levels of CPLX1 (*A–C*), CPLX2 (*D–F*), and SYNGR1 (*G–I*). Scale bar is 100 μm.

Out of many differentially expressed proteins, we selected a few candidate proteins for immunohistochemistry experiments based on the following parameters: (1) the fold change between AD and control (with a cut-off of 30% up- or down-regulation), (2) biological function, (3) proteins previously not studied in AD brain and (4) whether antibodies suitable for immunohistochemistry were commercially available. Finally, (5) we considered the fact that the region of our interest is highly enriched in excitatory synapses and that our comprehensive data analysis indicated an impaired presynaptic signaling. Using these criteria, we selected CPLX1, CPLX2, and SYNGR1 for further validation and performed immunohistochemistry in a new cohort consisting of five AD cases and seven controls. All the selected antibodies showed a strong immunoreactivity in the neuropil of the ML of the DG. CPLX2 also showed strong staining in the granule cell layer, whereas CPLX1 and SYNGR1 had a weak staining intensity in this region (Fig. 4). The mean pixel intensities of the outer two-thirds of the ML of the DG were measured using ImageJ Fiji (NIH) and the background, consisting of the mean pixel intensity of an area outside the tissue section, was subtracted. Semi-quantitative analysis showed 28% decrease for CPLX1 (*p* = 0.005, Fig. 4*A*–4*C*), 12% decreased for CPLX2 (*p* = 0.048, Fig. 4*D*–4*F*), and 26% decrease for SYNGR1 (*p* = 0.005, Fig. 4*G*–4*I*) in AD compared with control. As a further confirmation, subjective scoring done in a blinded fashion by two independent researchers (C.E. and S.F.) also indicated reduction in all three presynaptic proteins (data not shown).

## DISCUSSION

In this explorative study, we performed a MS-based proteomics to identify proteins that could play a role in synaptic dysfunction in AD. For this purpose, we focused on the outer two-thirds of the ML of the DG, which receives the main glutamatergic input of the hippocampus via the perforant path and importantly these synapses appear to be vulnerable to AD pathology. The use of HiRIEF prior to LC-MS (32) allowed for generation of in-depth MS-data, resulting in the identification of 7322 proteins, thus covering a significant proportion of the human proteome and enabling detection of alterations in low-abundant proteins of importance for disease pathogenesis.

**Fig. 5.**
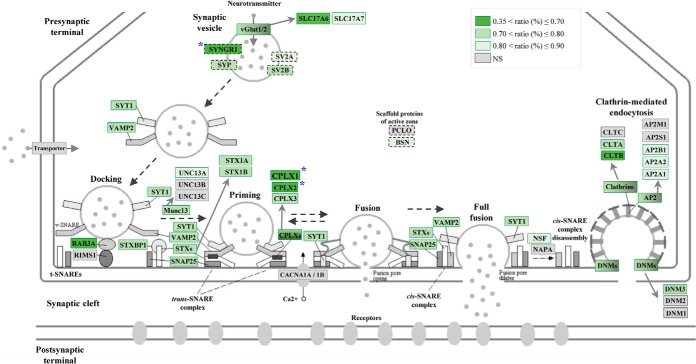
**Schematic overview of presynaptic proteins that were altered in AD.** The “synaptic vesicle pathway” (hsa04721) was modified from KEGG (66), and the proteins with altered expression in AD in our MS dataset were highlighted in different shades of green according to their ratio. The proteins with dashed line were added to the original pathway.

To gain insight into the potential mechanisms that could be affected by AD pathogenesis, we performed comprehensive enrichment and pathway analyses. GSEA, in which all proteins were analyzed based on their fold change, suggested an impaired mitochondrial function in the outer two-thirds of the ML of the DG in AD in accordance with the well-known impaired mitochondrial bioenergetics and increased oxidative stress in AD (2). Although many of the alterations of these individual mitochondrial proteins were not statistically significant, the large number of decreased mitochondrial proteins, particularly components of complex I, III, and IV, which were found to be slightly decreased, could still have an impact on mitochondrial function. Considering that synaptic transmission requires high amount of energy, altered mitochondrial bioenergetics could impair synaptic homeostasis, which has been previously observed in AD (44). To find out which biological processes are specifically enriched among the altered proteins (FDR <10%), we performed a second enrichment analysis using GOrilla tool. We found that neurotransmission secretion, calcium ion regulated exocytosis, and SV docking were negatively affected in the region of interest in AD. In line with this, pathway analysis using IPA also suggested an inhibition of exocytosis and secretory pathway in AD and created a molecular network which was enriched in presynaptic proteins. All together, these analyses strongly support a presynaptic impairment in the outer two-thirds of the ML of the DG in AD. Because synaptic loss is observed in this region of interest early in disease course (15, 16) which is thought to reflect the loss of afferents originating from the entorhinal cortex (13), it may not be surprising that many presynaptic proteins are decreased in our study. However, it is important to note that we did not observe any alterations in the postsynaptic density proteins (*e.g.* AMPA and NMDA receptors, PSD95) as well as majority of axonal transport proteins (*e.g.* KIF5B, KIF5C, DYNC1H1), indicating a specific loss of presynaptic proteins. Interestingly, several studies have shown increased postsynaptic apposition length of the remaining synapses in this region in AD (15, 16), which could be a mechanism to compensate for the reduced presynaptic input (45). Increasing evidence suggests that not all presynaptic proteins are affected in AD (46). In line with this finding, in our study, some presynaptic proteins showed minimal changes in AD whereas others were decreased to a greater degree (> 30%), as exemplified by CPLX1, CPLX2, vesicular glutamate transporter 2 (SLC17A6), SYNGR1, RAB3B, NCS1, SYNGR3, GRM4, and RAB3A (Fig. 5). Thus, some specific proteins are likely to be more important for proper presynaptic function than others. Even though many of the above-mentioned proteins have previously been shown to be presynaptic, we cannot rule out the possibility that they might be also expressed at the postsynaptic terminal. For further validation experiments, we selected three proteins (CPLX1, CPLX2, SYNGR1) that are presynaptic. Complexins interact with SNARE proteins and synaptotagmins and are important regulators of SV fusion (47). In support of our findings, reduced levels of CPLX1 and CPLX2 has been previously observed in the superior temporal cortex (48), in the hippocampus and the inferior temporal cortex of AD cases (49). SYNGR1 is an abundant protein that is expressed on SVs. Although its role remains to be elucidated, SYNGR1 is thought to modulate SV cycle and affect neurotransmitter release properties (50). SYNGR1 has not been studied extensively in postmortem brain tissue, but decreased levels of SYNGR1 has been detected in temporal cortex (22) and in CA1 of AD cases (51). We confirmed the decreased expression of CPLX1, CPLX2 and SYNGR1 in another cohort of AD and control cases, using immunohistochemistry.

Pathway analysis by IPA suggested involvement of processes related to seizures, ataxia and tremor processes based on the expression profile of the relevant proteins that were previously associated with these processes in the literature. Early hyperactivity has been detected in the medial temporal lobe in subjects with presymptomatic familial AD and mild cognitive impairment (52, 53), which could explain why seizures are observed in AD patients (54). Additionally, motor signs such as tremor and bradykinesia can accompany AD (55) and be indicative of worsening of the cognitive and especially the functional decline (56). Interestingly, phosphorylation of proteins was also found to be increased in AD. For example, calcineurin subunit B (PPP3R1) and serine/threonine protein phosphatase 2A (PP2CA), which both have been shown to dephosphorylate tau (57, 58), showed decreased expression in AD, in agreement with the presence of neurofibrillary tangles in this area. Enrichment analyses done by GSEA and GOrilla showed an activation of biological processes that are involved in regulation of gene expression, translational initiation, ribosome biogenesis, and RNA processing. These processes contain number of ribosomal proteins (*e.g.* hnRNPs, RPLs, RPSs) and eukaryotic translation initiation factors. In contrast to our findings, many ribosomal proteins have been previously shown to be decreased in AD brain (59, 60). These discrepancies could occur because of the local alterations in our region of interest. Activation of cell spreading and shape change of tumor cell lines as well as cell survival processes are perhaps surprising in a neurodegeneration context. However, many of these proteins, *e.g.* GFAP, CD44, ITGB1, EGFR, and GAP43, are also involved in other processes and it is possible that IPA predicted the above-mentioned pathways because of a potential over-representation of published literature in the cancer field. IPA can also predict upstream regulators of the altered proteins. One such upstream regulator identified by IPA was Nrf2, which is a transcription factor that mediates cellular defense mechanisms. In response to oxidative stress, Nrf2 translocates into the nucleus and targets the genes that contain anti-oxidant response elements (61). Extensive research suggests a disruption of Nrf2-mediated transcription pathway in aging and neurodegeneration. Ramsey and colleagues have observed decreased levels of nuclear Nrf2 in hippocampus of AD cases (62), whereas increased level of Nrf2 but decreased levels of its target genes were found in a meta-analysis of PD and AD microarray studies (63). Given the important roles of Aβ and hyperphosphorylated tau in AD pathogenesis, it was also interesting that APP and MAPT were predicted as upstream regulators by IPA. However, no activation state was predicted and thus we can only conclude that there is a relation between APP/MAPT and the altered proteins and there is no consensus in the literature on how they affect each other.

Last, we compared our findings with other proteomic studies that were done using brain homogenates of different regions from subjects with AD. We observed that number of proteins were altered in a similar way, as exemplified by decreased levels of STX1A, SYNGR1, SV2A, and SEPT5 in the temporal neocortex (22); decreased STX1B, SNAP91, PACSIN1 and increased GFAP, MAOB, VIM, and AQP4 levels in the microdissected CA1 and subiculum (28); decreased level of LRFN2 in the prefrontal cortex (24). On the other hand, some alterations were unique for our study, indicating that some changes might occur globally in AD brain whereas others are region-specific. A potential limitation of our study, especially compared with studies in which synaptosomal preparations have been used (64, 65), is the fact that our samples, although enriched in excitatory synapses, most probably contain other structures, such as axons, dendrites, glial cells and interneurons like MOPP cells. On the other hand, our study provides extensive information on biological activities that were found to be affected in this very specific highly vulnerable area in AD. Furthermore, the high number of synaptic proteins identified in our study indicates that we indeed microdissected a synapse rich area. The small sample size is another limitation of this study. However, we observed good separation between AD and control cases in the PCA plot, suggesting that the observed alterations in protein levels most likely are driven by AD pathogenesis. Furthermore, the alterations of CPLX1, CPLX2 and SYNGR1 were confirmed in another cohort, using immunohistochemistry.

In conclusion, we studied the proteome of the dentate terminal zone of the highly vulnerable perforant path in postmortem human brain tissue. Our in-depth proteomic analysis indicates a presynaptic impairment in the region of interest in AD, affecting processes like exocytosis and SV docking and specific proteins like CPLX1, CPLX2 and SYNGR1. Our extensive proteomics data and bioinformatic analysis supports the notion that presynaptic signaling is indeed affected in early stages of AD, and therefore, targeting presynaptic proteins might be crucial for future therapeutic strategies to slow or halt cognitive decline.

## DATA AVAILABILITY

Quantitative information about both peptide and protein level has been deposited to the ProteomeXchange Consortium using the PRIDE repository (https://www.ebi.ac.uk/pride/archive/projects/PXD014557, dataset identifier: PXD014557). All data generated or analyzed during this study are included in this paper and its supplementary information files.

## Supplementary Material

Supplementary Table 1

Supplementary Table 2

Supplementary Table 3

Supplementary Table 4

Supplementary Table 5

Supplementary Table 6

Supplementary figures

Supplementary Table 7

Supplementary Table 8

Supplementary Table 9

Supplementary Table 10

## Footnotes

^1^ The abbreviations used are:

AD: Alzheimer disease
Aβ: amyloid β-peptide
APP: amyloid precursor protein
CA: cornu ammonis
CPLX: complexin
DEqMS: Differential expression analysis of quantitative mass spectrometry data
DG: dentate gyrus
FDR: false discovery rate
GCL: granule cell layer
GO: gene ontology
GSEA: gene set enrichment analysis
HiRIEF: high resolution isoelectric focusing
IML: inner molecular layer
IPA: ingenuity pathway analysis
LC-MS/MS: liquid chromatography-tandem mass spectrometry
LMD: laser microdissection
MAPT: microtubule-associated protein tau
MML: middle molecular layer
ML: molecular layer
MOPP: molecular layer perforant path-associated
m/z: mass to charge ratio
OML: outer molecular layer
PCA: principal component analysis
PMI: postmortem interval
SV: synaptic vesicle
SYNGR1: synaptogyrin-1
TMT: tandem mass tag.

## References

[1] Jack C. R. Jr, Bennett D. A., Blennow K., Carrillo M. C., Dunn B., Haeberlein S. B., Holtzman D.M., Jagust W., Jessen F., Karlawish J., Liu E., Molinuevo J. L., Montine T., Phelps C., Rankin K. P., Rowe C.C., Scheltens P., Siemers E., Snyder H. M., Sperling R., and Contributors. (2018) NIA-AA Research Framework: Toward a biological definition of Alzheimer's disease. Alzheimers Dement. 14, 535–562 29653606 10.1016/j.jalz.2018.02.018 PMC5958625

[2] DuBoff B., Feany M., and Gotz J. (2013) Why size matters - balancing mitochondrial dynamics in Alzheimer's disease. Trends Neurosci. 36, 325–335 23582339 10.1016/j.tins.2013.03.002

[3] Nixon R. A., and Yang D. S. (2011) Autophagy failure in Alzheimer's disease–locating the primary defect. Neurobiol. Dis. 43, 38–45 21296668 10.1016/j.nbd.2011.01.021 PMC3096679

[4] Wang R., and Reddy P. H. (2017) Role of Glutamate and NMDA Receptors in Alzheimer's Disease. J. Alzheimers Dis. 57, 1041–1048 27662322 10.3233/JAD-160763 PMC5791143

[5] Heneka M. T., Carson M. J., El Khoury J., Landreth G. E., Brosseron F., Feinstein D. L., Jacobs A. H., Wyss-Coray T., Vitorica J., Ransohoff R. M., Herrup K., Frautschy S. A., Finsen B., Brown G. C., Verkhratsky A., Yamanaka K., Koistinaho J., Latz E., Halle A., Petzold G. C., Town T., Morgan D., Shinohara M. L., Perry V. H., Holmes C., Bazan N. G., Brooks D. J., Hunot S., Joseph B., Deigendesch N., Garaschuk O., Boddeke E., Dinarello C. A., Breitner J. C., Cole G. M., Golenbock D. T., and Kummer M. P. (2015) Neuroinflammation in Alzheimer's disease. Lancet Neurol. 14, 388–405 25792098 10.1016/S1474-4422(15)70016-5 PMC5909703

[6] DeKosky S. T., and Scheff S. W. (1990) Synapse loss in frontal cortex biopsies in Alzheimer's disease: correlation with cognitive severity. Ann. Neurol. 27, 457–464 2360787 10.1002/ana.410270502

[7] Bereczki E., Francis P. T., Howlett D., Pereira J. B., Hoglund K., Bogstedt A., Cedazo-Minguez A., Baek J. H., Hortobágyi T., Attems J., Ballard C., and Aarsland D. (2016) Synaptic proteins predict cognitive decline in Alzheimer's disease and Lewy body dementia. Alzheimers Dement. 12, 1149–1158 27224930 10.1016/j.jalz.2016.04.005

[8] Masliah E., Mallory M., Alford M., DeTeresa R., Hansen L. A., McKeel D. W. Jr, and Morris J. C. (2001) Altered expression of synaptic proteins occurs early during progression of Alzheimer's disease. Neurology 56, 127–129 11148253 10.1212/wnl.56.1.127

[9] Cappaert N. L. M., Van Strien N. M., and Witter M. P. (2015) Chapter 20 - Hippocampal Formation. In: Paxinos G, editor. The Rat Nervous System (Fourth Edition). San Diego: Academic Press; p. 511–73

[10] Amaral D. G., Scharfman H. E., and Lavenex P. (2007) The dentate gyrus: fundamental neuroanatomical organization (dentate gyrus for dummies). Prog. Brain Res. 163, 3–22 17765709 10.1016/S0079-6123(07)63001-5 PMC2492885

[11] Thal D. R., Holzer M., Rub U., Waldmann G., Gunzel S., Zedlick D., and Schober R. (2000) Alzheimer-related tau-pathology in the perforant path target zone and in the hippocampal stratum oriens and radiatum correlates with onset and degree of dementia. Exp. Neurol. 163, 98–110 10785448 10.1006/exnr.2000.7380

[12] Hyman B. T., Van Hoesen G. W., Kromer L. J., and Damasio A. R. (1986) Perforant pathway changes and the memory impairment of Alzheimer's disease. Ann. Neurol. 20, 472–481 3789663 10.1002/ana.410200406

[13] Gomez-Isla T., Price J. L., McKeel D. W. Jr, Morris J. C., Growdon J. H., and Hyman B. T. (1996) Profound loss of layer II entorhinal cortex neurons occurs in very mild Alzheimer's disease. J. Neurosci. 16, 4491–4500 8699259 10.1523/JNEUROSCI.16-14-04491.1996 PMC6578866

[14] Kordower J. H., Chu Y., Stebbins G. T., DeKosky S. T., Cochran E. J., Bennett D., and Mufson E. J. (2001) Loss and atrophy of layer II entorhinal cortex neurons in elderly people with mild cognitive impairment. Ann. Neurol. 49, 202–213 11220740

[15] Scheff S. W., Sparks D. L., and Price D. A. (1996) Quantitative assessment of synaptic density in the outer molecular layer of the hippocampal dentate gyrus in Alzheimer's disease. Dementia 7, 226–232 8835888 10.1159/000106884

[16] Scheff S. W., Price D. A., Schmitt F. A., and Mufson E. J. (2006) Hippocampal synaptic loss in early Alzheimer's disease and mild cognitive impairment. Neurobiol. Aging 27, 1372–1384 16289476 10.1016/j.neurobiolaging.2005.09.012

[17] Sheng M., and Kim E. (2011) The postsynaptic organization of synapses. Cold Spring Harb Perspect Biol. 3, a005678 22046028 10.1101/cshperspect.a005678 PMC3225953

[18] Sudhof T. C. (2013) Neurotransmitter release: the last millisecond in the life of a synaptic vesicle. Neuron 80, 675–690 24183019 10.1016/j.neuron.2013.10.022 PMC3866025

[19] Mufson E. J., Mahady L., Waters D., Counts S. E., Perez S. E., DeKosky S. T., Ginsberg S. D., Ikonomovic M. D., Scheff S. W., and Binder L. I. (2015) Hippocampal plasticity during the progression of Alzheimer's disease. Neuroscience 309, 51–67 25772787 10.1016/j.neuroscience.2015.03.006 PMC4567973

[20] de Wilde M. C., Overk C. R., Sijben J. W., and Masliah E. (2016) Meta-analysis of synaptic pathology in Alzheimer's disease reveals selective molecular vesicular machinery vulnerability. Alzheimers Dement. 12, 633–644 26776762 10.1016/j.jalz.2015.12.005 PMC5058345

[21] Manavalan A., Mishra M., Feng L., Sze S. K., Akatsu H., and Heese K. (2013) Brain site-specific proteome changes in aging-related dementia. Exp Mol. Med. 45, e39 24008896 10.1038/emm.2013.76 PMC3789264

[22] Musunuri S., Wetterhall M., Ingelsson M., Lannfelt L., Artemenko K., Bergquist J., Kultima K., and Shevchenko G. (2014) Quantification of the brain proteome in Alzheimer's disease using multiplexed mass spectrometry. J. Proteome Res. 13, 2056–2068 24606058 10.1021/pr401202d

[23] Seyfried N. T., Dammer E. B., Swarup V., Nandakumar D., Duong D. M., Yin L., Deng Q., Nguyen T., Hales C. M., Wingo T., Glass J., Gearing M., Thambisetty M., Troncoso J. C., Geschwind D. H., Lah J. J., and Levey A. I. (2017) A multi-network approach identifies protein-specific co-expression in asymptomatic and symptomatic Alzheimer's Disease. Cell systems 4, 60–72.e4 27989508 10.1016/j.cels.2016.11.006 PMC5269514

[24] Bereczki E., Branca R. M., Francis P. T., Pereira J. B., Baek J. H., Hortobagyi T., Winblad B., Ballard C., Lehtiö J., and Aarsland D. (2018) Synaptic markers of cognitive decline in neurodegenerative diseases: a proteomic approach. Brain 141, 582–595 29324989 10.1093/brain/awx352 PMC5837272

[25] Xu J., Patassini S., Rustogi N., Riba-Garcia I., Hale B. D., Phillips A. M., Waldvogel H., Haines R., Bradbury P., Stevens A., Faull R. L. M., Dowsey A. W., Cooper G. J. S., and Unwin R. D. (2019) Regional protein expression in human Alzheimer's brain correlates with disease severity. Communications Biol. 2, 43 10.1038/s42003-018-0254-9 PMC6361956 30729181

[26] Hashimoto M., Bogdanovic N., Nakagawa H., Volkmann I., Aoki M., Winblad B., and Tjernberg L. O. (2012) Analysis of microdissected neurons by 18O mass spectrometry reveals altered protein expression in Alzheimer's disease. J. Cell. Mol. Med. 16, 1686–1700 21883897 10.1111/j.1582-4934.2011.01441.x PMC3822682

[27] Ho Kim J., Franck J., Kang T., Heinsen H., Ravid R., Ferrer I., Hee Cheon M., Lee J. Y., Shin Yoo J., Steinbusch H. W., Salzet M., Fournier I., and Mok Park Y. (2015) Proteome-wide characterization of signalling interactions in the hippocampal CA4/DG subfield of patients with Alzheimer's disease. Sci. Rep. 5, 11138 26059363 10.1038/srep11138 PMC4462342

[28] Hondius D. C., van Nierop P., Li K. W., Hoozemans J. J., van der Schors R. C., van Haastert E. S., van der Vies S. M., Rozemuller A. J., and Smit A. B. (2016) Profiling the human hippocampal proteome at all pathologic stages of Alzheimer's disease. Alzheimers Dement. 12, 654–668 26772638 10.1016/j.jalz.2015.11.002

[29] Dubois B., Feldman H. H., Jacova C., Dekosky S. T., Barberger-Gateau P., Cummings J., Delacourte A., Galasko D., Gauthier S., Jicha G., Meguro K., O'brien J., Pasquier F., Robert P., Rossor M., Salloway S., Stern Y., Visser P. J., and Scheltens P. (2007) Research criteria for the diagnosis of Alzheimer's disease: revising the NINCDS-ADRDA criteria. Lancet Neurol. 6, 734–746 17616482 10.1016/S1474-4422(07)70178-3

[30] Montine T. J., Phelps C. H., Beach T. G., Bigio E. H., Cairns N. J., Dickson D. W., Duyckaerts C., Frosch M. P., Masliah E., Mirra S. S., Nelson P. T., Schneider J. A., Thal D. R., Trojanowski J. Q., Vinters H. V., Hyman B. T., and National Institute on Aging-Alzheimer's Association. (2012) National Institute on Aging-Alzheimer's Association guidelines for the neuropathologic assessment of Alzheimer's disease: a practical approach. Acta Neuropathol. 123, 1–11 22101365 10.1007/s00401-011-0910-3 PMC3268003

[31] Hughes C. S., Foehr S., Garfield D. A., Furlong E. E., Steinmetz L. M., and Krijgsveld J. (2014) Ultrasensitive proteome analysis using paramagnetic bead technology. Mol. Syst. Biol. 10, 757 25358341 10.15252/msb.20145625 PMC4299378

[32] Branca R. M., Orre L. M., Johansson H. J., Granholm V., Huss M., Perez-Bercoff A., Forshed J., Käll L., and Lehtiö J. (2014) HiRIEF LC-MS enables deep proteome coverage and unbiased proteogenomics. Nat. Methods 11, 59–62 24240322 10.1038/nmeth.2732

[33] Holman J. D., Tabb D. L., and Mallick P. (2014) Employing ProteoWizard to convert raw mass spectrometry data. Current Protocols Bioinformatics 46, 13.24.1–9 10.1002/0471250953.bi1324s46 PMC4113728 24939128

[34] Kim S., and Pevzner P. A. (2014) MS-GF+ makes progress towards a universal database search tool for proteomics. Nat. Communications 5, 5277 10.1038/ncomms6277 PMC5036525 25358478

[35] Granholm V., Kim S., Navarro J. C., Sjolund E., Smith R. D., and Kall L. (2014) Fast and accurate database searches with MS-GF+Percolator. J. Proteome Res. 13, 890–897 24344789 10.1021/pr400937n PMC3975676

[36] Boekel J., Chilton J. M., Cooke I. R., Horvatovich P. L., Jagtap P. D., Käll L., Lehtiö J., Lukasse P., Moerland P. D., and Griffin T. J. (2015) Multi-omic data analysis using Galaxy. Nat. Biotechnol. 33, 137 25658277 10.1038/nbt.3134

[37] Sturm M., Bertsch A., Gropl C., Hildebrandt A., Hussong R., Lange E., Pfeifer N., Schulz-Trieglaff O., Zerck A., Reinert K., and Kohlbacher O. (2008) OpenMS - an open-source software framework for mass spectrometry. BMC bioinformatics. 9, 163 18366760 10.1186/1471-2105-9-163 PMC2311306

[38] Savitski M. M., Wilhelm M., Hahne H., Kuster B., and Bantscheff M. (2015) A scalable approach for protein false discovery rate estimation in large proteomic data sets. Mol. Cell. Proteomics 14, 2394–2404 25987413 10.1074/mcp.M114.046995 PMC4563723

[39] Huber W., Carey V., Davis S., Hansen K., and Morgan M. (2016) The Bioconductor channel in F1000Research [version 2; peer review: not peer reviewed]. F1000Research. 4, 217 10.12688/f1000research.6758.1 PMC4786899 26998224

[40] Subramanian A., Tamayo P., Mootha V. K., Mukherjee S., Ebert B. L., Gillette M. A., et al (2005) Gene set enrichment analysis: a knowledge-based approach for interpreting genome-wide expression profiles. Proc. Natl. Acad. Sci. U.S.A. 102, 15545–15550 16199517 10.1073/pnas.0506580102 PMC1239896

[41] Liberzon A., Subramanian A., Pinchback R., Thorvaldsdottir H., Tamayo P., and Mesirov J. P. (2011) Molecular signatures database (MSigDB) 3.0. Bioinformatics (Oxford, England). 27, 1739–1740 10.1093/bioinformatics/btr260 PMC3106198 21546393

[42] Eden E., Navon R., Steinfeld I., Lipson D., and Yakhini Z. (2009) GOrilla: a tool for discovery and visualization of enriched GO terms in ranked gene lists. BMC Bioinformatics. 10, 48 19192299 10.1186/1471-2105-10-48 PMC2644678

[43] Robinson J. L., Molina-Porcel L., Corrada M. M., Raible K., Lee E. B., Lee V. M., Kawas C. H., and Trojanowski J. Q. (2014) Perforant path synaptic loss correlates with cognitive impairment and Alzheimer's disease in the oldest-old. Brain. 137, 2578–2587 25012223 10.1093/brain/awu190 PMC4132652

[44] Devine M. J., and Kittler J. T. (2018) Mitochondria at the neuronal presynapse in health and disease. Nat. Rev. Neurosci. 19, 63 10.1038/nrn.2017.170 29348666

[45] Hyman B. T., Van Hoesen G. W., and Damasio A. R. (1987) Alzheimer's disease: glutamate depletion in the hippocampal perforant pathway zone. Ann. Neurol. 22, 37–40 2443073 10.1002/ana.410220110

[46] Honer W. G. (2003) Pathology of presynaptic proteins in Alzheimer's disease: more than simple loss of terminals. Neurobiol. Aging 24, 1047–1062 14643376 10.1016/j.neurobiolaging.2003.04.005

[47] Gong J., Lai Y., Li X., Wang M., Leitz J., Hu Y., Zhang Y., Choi U. B., Cipriano D., Pfuetzner R. A., Südhof T. C., Yang X., Brunger A. T., and Diao J. (2016) C-terminal domain of mammalian complexin-1 localizes to highly curved membranes. Proc. Natl. Acad. Sci. U.S.A. 113, E7590–E7599 27821736 10.1073/pnas.1609917113 PMC5127347

[48] Beeri M. S., Haroutunian V., Schmeidler J., Sano M., Fam P., Kavanaugh A., Barr A. M., Honer W. G., and Katsel P. (2012) Synaptic protein deficits are associated with dementia irrespective of extreme old age. Neurobiol. Aging 33, 1125.e1–8 10.1016/j.neurobiolaging.2011.08.017 PMC3318958 22206847

[49] Tannenberg R. K., Scott H. L., Tannenberg A. E., and Dodd P. R. (2006) Selective loss of synaptic proteins in Alzheimer's disease: evidence for an increased severity with APOE varepsilon4. Neurochem. Int. 49, 631–639 16814428 10.1016/j.neuint.2006.05.004

[50] Stevens R. J., Akbergenova Y., Jorquera R. A., Littleton J. T. (2012) Abnormal synaptic vesicle biogenesis in Drosophila synaptogyrin mutants. J. Neurosci. 32, 18054–18067, 67a 23238721 10.1523/JNEUROSCI.2668-12.2012 PMC3530745

[51] Counts S. E., Alldred M. J., Che S., Ginsberg S. D., and Mufson E. J. (2014) Synaptic gene dysregulation within hippocampal CA1 pyramidal neurons in mild cognitive impairment. Neuropharmacology 79, 172–179 24445080 10.1016/j.neuropharm.2013.10.018 PMC3951099

[52] Quiroz Y. T., Budson A. E., Celone K., Ruiz A., Newmark R., Castrillon G., Lopera F., and Stern C. E. (2010) Hippocampal hyperactivation in presymptomatic familial Alzheimer's disease. Ann. Neurol. 68, 865–875 21194156 10.1002/ana.22105 PMC3175143

[53] Putcha D., Brickhouse M., O'Keefe K., Sullivan C., Rentz D., Marshall G., Dickerson B., and Sperling R. (2011) Hippocampal hyperactivation associated with cortical thinning in Alzheimer's disease signature regions in non-demented elderly adults. J. Neurosci. 31, 17680–17688 22131428 10.1523/JNEUROSCI.4740-11.2011 PMC3289551

[54] Irizarry M. C., Jin S., He F., Emond J. A., Raman R., Thomas R. G., Sano M., Quinn J. F., Tariot P. N., Galasko D. R., Ishihara L. S., Weil J. G., and Aisen P. S. (2012) Incidence of new-onset seizures in mild to moderate Alzheimer disease. Arch. Neurol. 69, 368–372 22410444 10.1001/archneurol.2011.830 PMC3622046

[55] Ellis R. J., Caligiuri M., Galasko D., and Thal L. J. (1996) Extrapyramidal motor signs in clinically diagnosed Alzheimer disease. Alzheimer Dis. Assoc. Disorders 10, 103–114 10.1097/00002093-199601020-00008 8727172

[56] Scarmeas N., Albert M., Brandt J., Blacker D., Hadjigeorgiou G., Papadimitriou A., Dubois B., Sarazin M., Wegesin D., Marder K., Bell K., Honig L., and Stern Y. (2005) Motor signs predict poor outcomes in Alzheimer disease. Neurology 64, 1696–1703 15911793 10.1212/01.WNL.0000162054.15428.E9 PMC3028937

[57] Yamamoto H., Saitoh Y., Fukunaga K., Nishimura H., and Miyamoto E. (1988) Dephosphorylation of microtubule proteins by brain protein phosphatases 1 and 2A, and its effect on microtubule assembly. J. Neurochem. 50, 1614–1623 2834518 10.1111/j.1471-4159.1988.tb03051.x

[58] Goto S., Yamamoto H., Fukunaga K., Iwasa T., Matsukado Y., and Miyamoto E. (1985) Dephosphorylation of microtubule-associated protein 2, tau factor, and tubulin by calcineurin. J. Neurochem. 45, 276–283 2987415 10.1111/j.1471-4159.1985.tb05504.x

[59] Hernandez-Ortega K., Garcia-Esparcia P., Gil L., Lucas J. J., and Ferrer I. (2016) Altered machinery of protein synthesis in Alzheimer's: from the nucleolus to the ribosome. Brain Pathol. 26, 593–605 26512942 10.1111/bpa.12335 PMC8029302

[60] Berson A., Barbash S., Shaltiel G., Goll Y., Hanin G., Greenberg D. S., Ketzef M., Becker A. J., Friedman A., Soreq H. (2012) Cholinergic-associated loss of hnRNP-A/B in Alzheimer's disease impairs cortical splicing and cognitive function in mice. EMBO Mol. Med. 4, 730–742 22628224 10.1002/emmm.201100995 PMC3494073

[61] Lee J. M., and Johnson J. A. (2004) An important role of Nrf2-ARE pathway in the cellular defense mechanism. J. Biochem. Mol. Biol. 37, 139–143 15469687 10.5483/bmbrep.2004.37.2.139

[62] Ramsey C. P., Glass C. A., Montgomery M. B., Lindl K. A., Ritson G. P., Chia L. A., Hamilton R. L., Chu C. T., and Jordan-Sciutto K. L. (2007) Expression of Nrf2 in neurodegenerative diseases. J. Neuropathol. Exp. Neurol. 66, 75–85 17204939 10.1097/nen.0b013e31802d6da9 PMC2253896

[63] Wang Q., Li W. X., Dai S. X., Guo Y. C., Han F. F., Zheng J. J., Li G. H., Huang J. F. (2017) Meta-analysis of Parkinson's disease and Alzheimer's disease revealed commonly impaired pathways and dysregulation of NRF2-dependent genes. J. Alzheimers. Dis. 56, 1525–1539 28222515 10.3233/JAD-161032

[64] Zhou J., Jones D. R., Duong D. M., Levey A. I., Lah J. J., and Peng J. (2013) Proteomic analysis of postsynaptic density in Alzheimer's disease. Clin. Chim. Acta 420, 62–68 23537733 10.1016/j.cca.2013.03.016 PMC3714371

[65] Chang R. Y., Nouwens A. S., Dodd P. R., and Etheridge N. (2013) The synaptic proteome in Alzheimer's disease. Alzheimers Dement. 9, 499–511 23154051 10.1016/j.jalz.2012.04.009

[66] Kanehisa M., Sato Y., Furumichi M., Morishima K., and Tanabe M. (2019) New approach for understanding genome variations in KEGG. Nucleic Acids Res. 47, D590–D595 30321428 10.1093/nar/gky962 PMC6324070

