# Supplementary material for: The Proteome of the Dentate Terminal Zone of the Perforant Path Indicates Presynaptic Impairment in Alzheimer Disease
Source: Mol Cell Proteomics. 2019 Nov 7;19(1):128–41. doi: 10.1074/mcp.RA119.001737 (PMC6944231; doi:10.1074/mcp.RA119.001737)
Supplement: Supplementary Table 1 [file 155278_2_supp_424229_q0v4bp.pdf]

Supplementary Table 1. Clinical and neuropathological details about all cases used in this study.

Case	Diagnosis	Gender	Age	PMI	Brain pH	APOE status	Braak tau stage	Amyloid stage
**Exploratory cohort: LC-MS experiments**								
1	AD	F	95	3:40	6.86	4/3	4	C
2	AD	F	91	6:05	6.21	3/3	4	C
3	AD	F	91	6:25	6.05	3/3	4	C
4	AD	F	84	6:00	6.57	n/a	4	C
5	AD	M	94	4:15	7.11	n/a	4	C
6	C	F	72	6:40	6.07	4/2	0	n/a
7	C	F	72	8:15	5.81	3/3	0	0
8	C	F	92	6:55	6.24	4/3	2	B
9	C	M	88	5:40	6.17	n/a	1	n/a
10	C	F	85	7:50	6.09	n/a	2	A
	**p-value:**	**NS**	**NS**	**NS**	**NS**	**-**	**0.007**	**0.01**
**Verification cohort: IHC experiments**								
1	EOAD	F	63	14	n/a	3/4	6	C
2	EOAD	F	55	8.2	n/a	3/4	4	C
3	AD	M	76	6.5	n/a	4/4	4-5	C
4	AD	F	74	6.5	n/a	4/4	4-5	C
5	AD	F	85	4.7	n/a	3/4	5-6	C
6	C	F	100	11.5	n/a	3/3	1	B
7	C	M	81	8	n/a	3/3	2	0
8	C	F	86	5.5	n/a	3/3	1-2	A
9	C	M	61	16	n/a	2/3	2	0
10	C	M	59	16.2	n/a	3/3	2	0
11	C	F	80	7	n/a	3/3	2	0
12	C	M	67	12.2	n/a	3/3	0-2	0
	**p-value:**	**NS**	**NS**	**NS**	**-**	**0.001**	**0.001**	**0.002**

The clinical and pathological characteristics were compared between AD and control groups using Mann Whitney test, and $p < 0.05$ considered statistically significant.
