# Supplementary material for: The Proteome of the Dentate Terminal Zone of the Perforant Path Indicates Presynaptic Impairment in Alzheimer Disease
Source: Mol Cell Proteomics. 2019 Nov 7;19(1):128–41. doi: 10.1074/mcp.RA119.001737 (PMC6944231; doi:10.1074/mcp.RA119.001737)
Supplement: Supplementary figures [file 155278_2_supp_424238_q010qd.pdf]

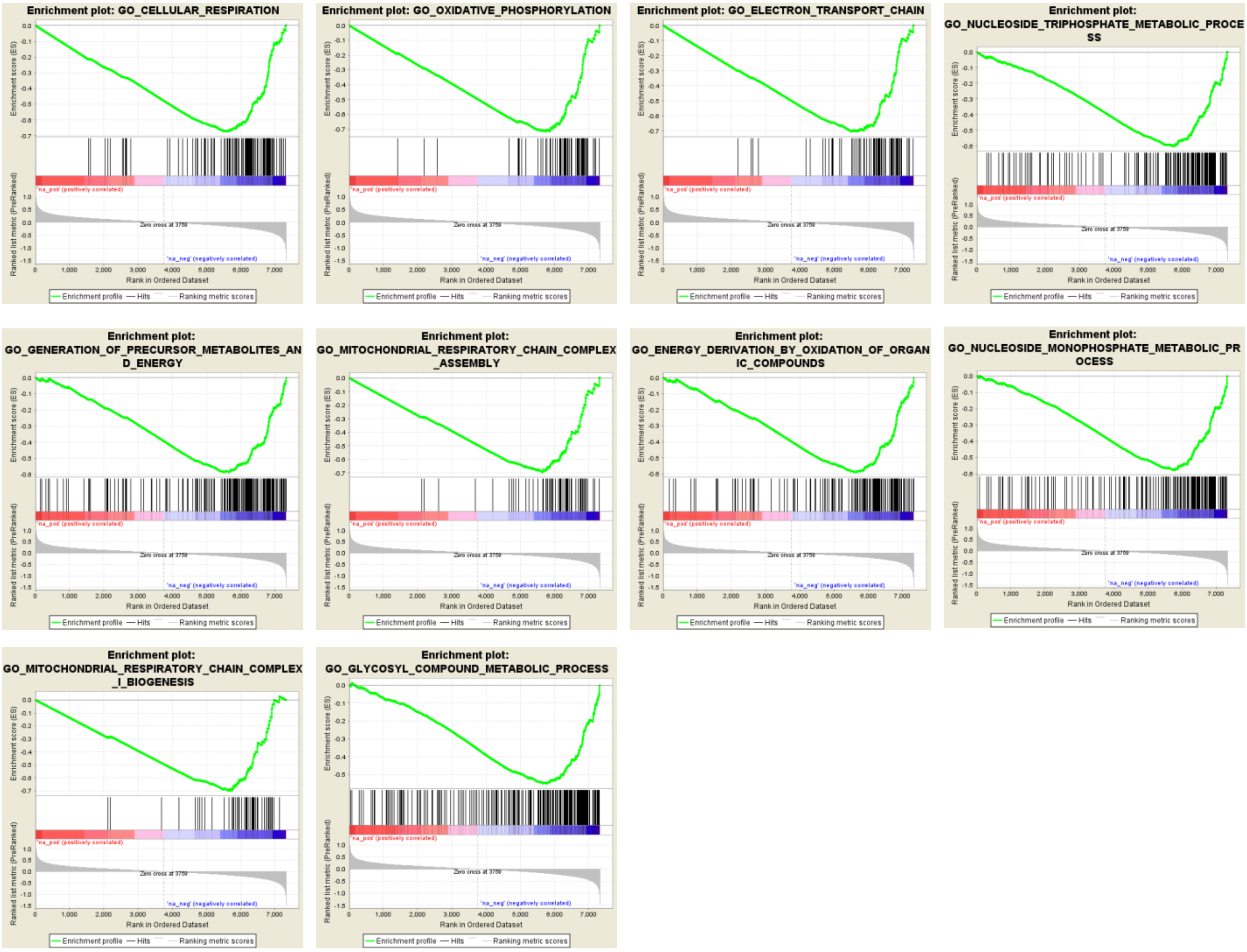


**Supplementary Fig. 1.** Individual enrichment plots of the top 10 negatively enriched biological process in the region of interest in AD.

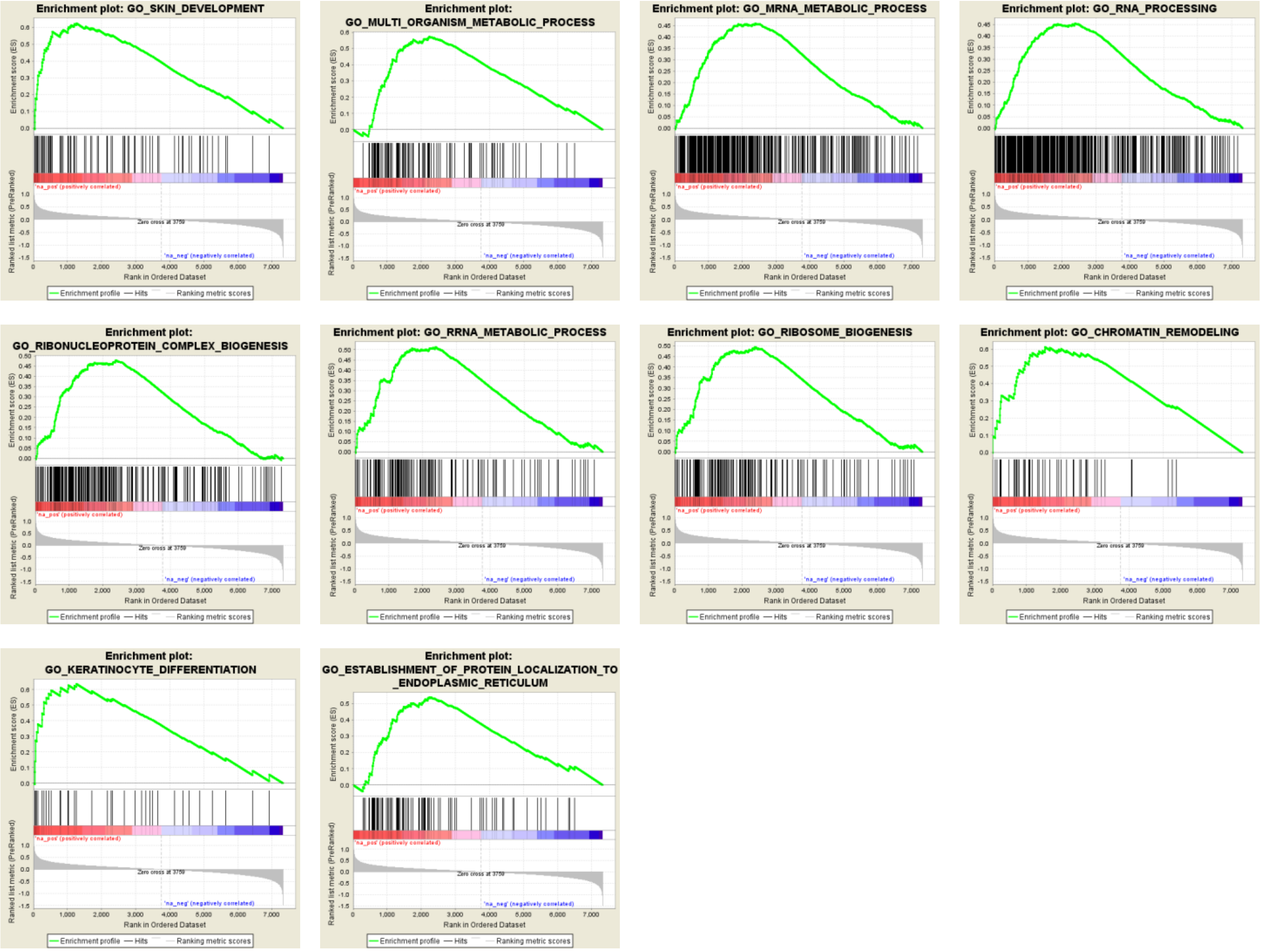


**Supplementary Fig. 2.** Individual enrichment plots of top 10 positively enriched biological process in the region of interest in AD.
