# Supplementary material for: The Proteome of the Dentate Terminal Zone of the Perforant Path Indicates Presynaptic Impairment in Alzheimer Disease
Source: Mol Cell Proteomics. 2019 Nov 7;19(1):128–41. doi: 10.1074/mcp.RA119.001737 (PMC6944231; doi:10.1074/mcp.RA119.001737)
Supplement: Supplementary Table 9 [file 155278_2_supp_424239_q0q0qd.pdf]

Supplementary Table 9. Biological functions that are predicted to be affected in AD are shown.

Diseases and Functions Annotation	Predicted activation state	Activation z-score	p-value	Target proteins in our dataset	# Proteins
Secretory pathway	Decreased	-2.570	2.44E-11	↑ ANXA1<br>↓ CADPS2, CPLX2, NCS1, RAB3A, RAB3B, RAB3D, SEPT5, SNAP25, SNAP29, STX1A, VSNL1	30
Exocytosis (GO:0006887, GO:0017157)	Decreased	-2.570	1.13E-10	↑ ANXA1<br>↓ CADPS2, CPLX2, NCS1, RAB3A, RAB3B, RAB3D, SEPT5, SNAP25, SNAP29, STX1A, VSNL1	28
Neuron migration[a] (GO:0001764)	Decreased	-2.528	3.55E-07	↑ CDK5R1, GNA12, GNA13, ITGB1<br>↓ CELSR3, DAB1, DCC, FSCN1, HSPA8, RTN4, SEMA4D, SEPT4, SLC12A2, PTPRZ1, STMN1, TIAM2	31
Migration of central nervous system cells	Decreased	-2.156	4.69E-05	↑ GNA12, GNA13, STAT3<br>↓ CELSR3, DAB1, SEPT4, TIAM2	13
Metal ion transport (GO:0030001)	Decreased	-2.619	4.44E-04	↓ AKT3, ANK3, DLG1, DPP6, FGF12, SCN2A, SCN3B	27
Seizures[b] (MPO:0002064, SnoMed:91175000)	Increased	2.888	5.29E-08	↓ AKT3, ALDH5A1, ANK3, BSN, CNTN2, CPLX1, GNAO1, GNG3, KCNA1, KCNA2, KCNMB4, PACSIN1, PIPTNA, PLCB1, PPP3CB, PTPRN2, SCG5, SCN2B, SLC9A1, SV2B	51
Cell spreading of tumor cell lines[c]	Increased	2.137	1.88E-07	↑ EGFR, GFAP, ITGB1, SPARC, SYNM<br>↓ GAP43, SIRPA	10
Ataxia[d] (MPO:0001393, SnoMed:20262006)	Increased	2.280	4.41E-07	↓ ALDH5A1, ANK3, APBA1, CAMK4, CNTNAP1, CPLX1, DAB1, DNAJC5, EPB41L3, KCNA1, NRCAM, PITPNA, SLC12A2, SLC9A1	34
Shape change of tumor cell lines	Increased	2.534	5.27E-07	↑ EGFR, GFAP, GNA12, GNA13, ITGB1, SPARC, SYNM<br>↓ GAP43, SIRPA	12
Phosphorylation of protein (GO:0001932, GO:0006468,GO:0016310, GO:0042325)	Increased	2.511	5.20E-05	↑ CD44, DCN, EGFR, ILK, ITGA6, KIT, MAP3K5, NCKAP1L, PDGFRB, SQSTM1, SYK<br>↓ CD55 CHP1, FKBP1A, HSPA4, LGALS1, MYADM, PPP2CA, WARS	55
Tremor[d] (MPO:0000745, SnoMed:26079004)	Increased	2.547	4.35E-04	↓ ADAM23, ANK3, CAMK4, CNTNAP1, CPE, GNAO1, KCNA1, MAG, PITPNA	17
Cell survival	Increased	3.149	8.87E-04	↑ CD151, CD44, CDC40, CHD4, CLNS1A, DDX3X, EGFR, HDAC1, ILK, ITGB1, KIT, NBN, PDGFRB, PNKP, PRKG1, RPS6KA2, SEL1L, SF3B1, SMC3, STAT3, SYNM<br>↓ CD55, ERBB3, FHIT, IGFBP2, LANCL2, LINGO1, MOG, NEFL	47

Differentially expressed proteins (n = 724, FDR <10%) were uploaded into the IPA and diseases and functions tool was used. Predictions about the activation state were based on the listed proteins. The p-value of overlap is calculated by right-tailed Fisher's Exact Test and p <0.01 considered statistically significant. The arrows indicate the identified proteins in our study having increased or decreased levels, respectively. Four ancestor GO terms that were also identified by IPA included [a] Cell movement of neurons, [b] Seizure disorder (SnoMed:128613002), [c] Cell spreading, and [d] Motor dysfunction or movement disorder (MPO:0002066).
