# Supplementary material for: The Proteome of the Dentate Terminal Zone of the Perforant Path Indicates Presynaptic Impairment in Alzheimer Disease
Source: Mol Cell Proteomics. 2019 Nov 7;19(1):128–41. doi: 10.1074/mcp.RA119.001737 (PMC6944231; doi:10.1074/mcp.RA119.001737)
Supplement: Supplementary Table 10 [file 155278_2_supp_424240_q050qd.pdf]

Supplementary Table 10. Predicted upstream regulators that could explain the altered levels of proteins identified in our data.

Upstream regulator	Predicted activation state	Activation z-score	p-value of overlap	Target proteins in our dataset	# Proteins
MAP kinase-interacting serine/threonine-protein kinase 1 (MKNK1)	**Inhibited**	-2.500	1.32E-06	↑ ARVCF, PLXNB2, SPARC<br>↓ ACOT7, CP, CPLX2, CRMP1, GAP43, GNAI1, GNAO1, HSPA4L, RAB14, RAB3A, SNAP25, VAMP2, VSNL1	16
Transcription factor 7-like 2 (TCF7L2)	**Inhibited**	-3.400	4.65E-05	↑ ERBIN, EVI2B, ILK, ROCK<br>↓ APLP1, BIN1, CNP, ERBB3, FKBP1A, FTH1, GLTP, IDH1, MAG, MOG, NINJ2, PIP4K2A, RAP2A, RHOA, RTKN, SEMA4D, SIRT2, SLC12A2, SLC22A23, TMEM163, TPPP	25
Haemoglobin subunit alpha (HBA1/HBA2)	**Inhibited**	-2.646	0.0016	↓ ATP5MF, NDUFA13, NDUFA6, NDUFA9, NDUFV1, UQCRC1, UQCRC2	7
Nuclear factor erythroid 2-related factor 2 (NFE2L2)	**Inhibited**	-2.525	0.0045	↑ GFAP, KEAP1<br>↓ CHGB, DCTN3, GNG3, KCNMB4, SLC7A8, SYP, SYT1, VSNL1	11

Differentially expressed proteins (n = 724, FDR <10%) were uploaded into the IPA and upstream analysis tool was used. MKNK1, TCF7L2, HBA1/HBA2, and NFE2L2 are all predicted to be inhibited by IPA. The p-value of overlap is calculated by right-tailed Fisher's Exact Test and p <0.01 considered statistically significant. The arrows indicate the identified proteins in our study having increased or decreased levels, respectively.
